# Supplementary material for: Multi‐dataset identification of innovative feature genes and molecular mechanisms in keratoconus
Source: J Cell Mol Med. 2024 Sep 19;28(18):e70079. doi: 10.1111/jcmm.70079 (PMC11412914; doi:10.1111/jcmm.70079)
Supplement: Supplementary file 2 — Material S2. [file JCMM-28-e70079-s002.zip › 7.GSEA analysis/4.MYRF/gsea_report_for_l_1678852873579.html]

Report for l 1678852873579 [GSEA]

| GS  follow link to MSigDB | GS DETAILS | SIZE | ES | NES | NOM p-val | FDR q-val | FWER p-val | RANK AT MAX | LEADING EDGE || 1 | KEGG\_EPITHELIAL\_CELL\_SIGNALING\_IN\_HELICOBACTER\_PYLORI\_INFECTION | Details ... | 66 | -0.55 | -1.92 | 0.006 | 0.048 | 0.033 | 3644 | tags=21%, list=10%, signal=24% |
| 2 | KEGG\_NOD\_LIKE\_RECEPTOR\_SIGNALING\_PATHWAY | Details ... | 61 | -0.53 | -1.74 | 0.036 | 0.309 | 0.289 | 4124 | tags=23%, list=12%, signal=26% |
| 3 | KEGG\_PATHWAYS\_IN\_CANCER | Details ... | 322 | -0.40 | -1.74 | 0.002 | 0.215 | 0.301 | 7064 | tags=25%, list=20%, signal=31% |
| 4 | KEGG\_BASAL\_CELL\_CARCINOMA | Details ... | 55 | -0.59 | -1.73 | 0.002 | 0.187 | 0.329 | 7064 | tags=44%, list=20%, signal=55% |
| 5 | KEGG\_MAPK\_SIGNALING\_PATHWAY | Details ... | 265 | -0.41 | -1.68 | 0.002 | 0.242 | 0.439 | 8505 | tags=32%, list=24%, signal=43% |
| 6 | KEGG\_TYROSINE\_METABOLISM | Details ... | 40 | -0.51 | -1.68 | 0.004 | 0.206 | 0.447 | 2223 | tags=25%, list=6%, signal=27% |
| 7 | KEGG\_TGF\_BETA\_SIGNALING\_PATHWAY | Details ... | 85 | -0.49 | -1.64 | 0.004 | 0.253 | 0.523 | 2580 | tags=21%, list=7%, signal=23% |
| 8 | KEGG\_BLADDER\_CANCER | Details ... | 41 | -0.56 | -1.64 | 0.029 | 0.222 | 0.523 | 7023 | tags=29%, list=20%, signal=37% |
| 9 | KEGG\_ARRHYTHMOGENIC\_RIGHT\_VENTRICULAR\_CARDIOMYOPATHY\_ARVC | Details ... | 74 | -0.52 | -1.63 | 0.010 | 0.215 | 0.538 | 7080 | tags=46%, list=20%, signal=58% |
| 10 | KEGG\_NEUROTROPHIN\_SIGNALING\_PATHWAY | Details ... | 125 | -0.33 | -1.61 | 0.027 | 0.228 | 0.579 | 6504 | tags=18%, list=19%, signal=23% |
| 11 | KEGG\_PROSTATE\_CANCER | Details ... | 89 | -0.37 | -1.59 | 0.012 | 0.257 | 0.632 | 4711 | tags=17%, list=14%, signal=19% |
| 12 | KEGG\_CHEMOKINE\_SIGNALING\_PATHWAY | Details ... | 183 | -0.45 | -1.59 | 0.011 | 0.236 | 0.632 | 6949 | tags=28%, list=20%, signal=35% |
| 13 | KEGG\_INTESTINAL\_IMMUNE\_NETWORK\_FOR\_IGA\_PRODUCTION | Details ... | 48 | -0.59 | -1.59 | 0.006 | 0.219 | 0.633 | 2677 | tags=35%, list=8%, signal=38% |
| 14 | KEGG\_GRAFT\_VERSUS\_HOST\_DISEASE | Details ... | 39 | -0.62 | -1.58 | 0.010 | 0.221 | 0.650 | 4919 | tags=38%, list=14%, signal=45% |
| 15 | KEGG\_ADHERENS\_JUNCTION | Details ... | 68 | -0.41 | -1.57 | 0.078 | 0.217 | 0.657 | 10006 | tags=34%, list=29%, signal=47% |
| 16 | KEGG\_CHRONIC\_MYELOID\_LEUKEMIA | Details ... | 73 | -0.39 | -1.57 | 0.070 | 0.210 | 0.664 | 7023 | tags=23%, list=20%, signal=29% |
| 17 | KEGG\_ACUTE\_MYELOID\_LEUKEMIA | Details ... | 57 | -0.37 | -1.57 | 0.052 | 0.199 | 0.665 | 7247 | tags=28%, list=21%, signal=35% |
| 18 | KEGG\_MELANOGENESIS | Details ... | 101 | -0.41 | -1.56 | 0.010 | 0.201 | 0.674 | 7775 | tags=32%, list=22%, signal=41% |
| 19 | KEGG\_TOLL\_LIKE\_RECEPTOR\_SIGNALING\_PATHWAY | Details ... | 96 | -0.45 | -1.55 | 0.017 | 0.202 | 0.689 | 5132 | tags=25%, list=15%, signal=29% |
| 20 | KEGG\_CELL\_ADHESION\_MOLECULES\_CAMS | Details ... | 130 | -0.47 | -1.53 | 0.012 | 0.216 | 0.713 | 5434 | tags=32%, list=16%, signal=37% |
| 21 | KEGG\_PRION\_DISEASES | Details ... | 35 | -0.53 | -1.53 | 0.022 | 0.211 | 0.720 | 4957 | tags=37%, list=14%, signal=43% |
| 22 | KEGG\_ADIPOCYTOKINE\_SIGNALING\_PATHWAY | Details ... | 66 | -0.42 | -1.52 | 0.052 | 0.209 | 0.731 | 7375 | tags=30%, list=21%, signal=38% |
| 23 | KEGG\_LEISHMANIA\_INFECTION | Details ... | 71 | -0.54 | -1.52 | 0.041 | 0.203 | 0.735 | 4928 | tags=37%, list=14%, signal=43% |
| 24 | KEGG\_GLYCOSAMINOGLYCAN\_BIOSYNTHESIS\_CHONDROITIN\_SULFATE | Details ... | 22 | -0.58 | -1.52 | 0.056 | 0.196 | 0.739 | 8075 | tags=41%, list=23%, signal=53% |
| 25 | KEGG\_CYTOKINE\_CYTOKINE\_RECEPTOR\_INTERACTION | Details ... | 256 | -0.50 | -1.50 | 0.010 | 0.215 | 0.763 | 7694 | tags=45%, list=22%, signal=57% |
| 26 | KEGG\_INOSITOL\_PHOSPHATE\_METABOLISM | Details ... | 54 | -0.38 | -1.50 | 0.072 | 0.212 | 0.766 | 10039 | tags=35%, list=29%, signal=49% |
| 27 | KEGG\_FC\_EPSILON\_RI\_SIGNALING\_PATHWAY | Details ... | 78 | -0.43 | -1.50 | 0.022 | 0.207 | 0.767 | 4711 | tags=21%, list=14%, signal=24% |
| 28 | KEGG\_RIG\_I\_LIKE\_RECEPTOR\_SIGNALING\_PATHWAY | Details ... | 64 | -0.39 | -1.49 | 0.022 | 0.205 | 0.772 | 3657 | tags=16%, list=11%, signal=17% |
| 29 | KEGG\_P53\_SIGNALING\_PATHWAY | Details ... | 66 | -0.40 | -1.49 | 0.080 | 0.204 | 0.776 | 10446 | tags=42%, list=30%, signal=61% |
| 30 | KEGG\_SMALL\_CELL\_LUNG\_CANCER | Details ... | 84 | -0.37 | -1.48 | 0.081 | 0.207 | 0.787 | 8941 | tags=35%, list=26%, signal=46% |
| 31 | KEGG\_HYPERTROPHIC\_CARDIOMYOPATHY\_HCM | Details ... | 83 | -0.43 | -1.47 | 0.020 | 0.212 | 0.801 | 5871 | tags=35%, list=17%, signal=42% |
| 32 | KEGG\_TIGHT\_JUNCTION | Details ... | 127 | -0.40 | -1.46 | 0.028 | 0.220 | 0.811 | 7557 | tags=27%, list=22%, signal=34% |
| 33 | KEGG\_WNT\_SIGNALING\_PATHWAY | Details ... | 149 | -0.34 | -1.46 | 0.029 | 0.217 | 0.815 | 7904 | tags=26%, list=23%, signal=34% |
| 34 | KEGG\_TYPE\_I\_DIABETES\_MELLITUS | Details ... | 43 | -0.54 | -1.45 | 0.060 | 0.220 | 0.821 | 4499 | tags=35%, list=13%, signal=40% |
| 35 | KEGG\_ASTHMA | Details ... | 29 | -0.66 | -1.45 | 0.078 | 0.215 | 0.823 | 4483 | tags=48%, list=13%, signal=55% |
| 36 | KEGG\_AXON\_GUIDANCE | Details ... | 128 | -0.38 | -1.45 | 0.026 | 0.212 | 0.826 | 4876 | tags=20%, list=14%, signal=24% |
| 37 | KEGG\_FOCAL\_ADHESION | Details ... | 196 | -0.41 | -1.45 | 0.084 | 0.212 | 0.829 | 8960 | tags=37%, list=26%, signal=50% |
| 38 | KEGG\_CELL\_CYCLE | Details ... | 124 | -0.42 | -1.44 | 0.130 | 0.208 | 0.830 | 10818 | tags=43%, list=31%, signal=62% |
| 39 | KEGG\_REGULATION\_OF\_ACTIN\_CYTOSKELETON | Details ... | 210 | -0.33 | -1.43 | 0.026 | 0.216 | 0.842 | 9623 | tags=34%, list=28%, signal=46% |
| 40 | KEGG\_HEDGEHOG\_SIGNALING\_PATHWAY | Details ... | 55 | -0.44 | -1.42 | 0.044 | 0.233 | 0.861 | 7215 | tags=35%, list=21%, signal=44% |
| 41 | KEGG\_SPHINGOLIPID\_METABOLISM | Details ... | 35 | -0.41 | -1.42 | 0.067 | 0.232 | 0.861 | 6540 | tags=26%, list=19%, signal=32% |
| 42 | KEGG\_ALLOGRAFT\_REJECTION | Details ... | 37 | -0.58 | -1.40 | 0.077 | 0.243 | 0.879 | 4483 | tags=38%, list=13%, signal=43% |
| 43 | KEGG\_BASAL\_TRANSCRIPTION\_FACTORS | Details ... | 33 | -0.33 | -1.40 | 0.120 | 0.237 | 0.879 | 12680 | tags=45%, list=37%, signal=72% |
| 44 | KEGG\_DILATED\_CARDIOMYOPATHY | Details ... | 90 | -0.38 | -1.39 | 0.039 | 0.255 | 0.894 | 5871 | tags=32%, list=17%, signal=39% |
| 45 | KEGG\_VIRAL\_MYOCARDITIS | Details ... | 70 | -0.44 | -1.39 | 0.119 | 0.250 | 0.894 | 5871 | tags=27%, list=17%, signal=33% |
| 46 | KEGG\_ETHER\_LIPID\_METABOLISM | Details ... | 29 | -0.55 | -1.38 | 0.098 | 0.259 | 0.901 | 1719 | tags=17%, list=5%, signal=18% |
| 47 | KEGG\_STEROID\_HORMONE\_BIOSYNTHESIS | Details ... | 46 | -0.45 | -1.38 | 0.033 | 0.257 | 0.903 | 5255 | tags=35%, list=15%, signal=41% |
| 48 | KEGG\_T\_CELL\_RECEPTOR\_SIGNALING\_PATHWAY | Details ... | 107 | -0.37 | -1.37 | 0.077 | 0.253 | 0.903 | 4795 | tags=20%, list=14%, signal=23% |
| 49 | KEGG\_GLYCOSAMINOGLYCAN\_BIOSYNTHESIS\_KERATAN\_SULFATE | Details ... | 15 | -0.49 | -1.37 | 0.140 | 0.248 | 0.904 | 7663 | tags=47%, list=22%, signal=60% |
| 50 | KEGG\_DORSO\_VENTRAL\_AXIS\_FORMATION | Details ... | 24 | -0.44 | -1.37 | 0.131 | 0.253 | 0.912 | 4580 | tags=21%, list=13%, signal=24% |
| 51 | KEGG\_ALZHEIMERS\_DISEASE |  | 144 | -0.26 | -1.36 | 0.104 | 0.259 | 0.921 | 6969 | tags=12%, list=20%, signal=15% |
| 52 | KEGG\_PANCREATIC\_CANCER |  | 69 | -0.31 | -1.36 | 0.118 | 0.258 | 0.924 | 6489 | tags=17%, list=19%, signal=21% |
| 53 | KEGG\_INSULIN\_SIGNALING\_PATHWAY |  | 136 | -0.31 | -1.35 | 0.136 | 0.258 | 0.928 | 7287 | tags=21%, list=21%, signal=27% |
| 54 | KEGG\_APOPTOSIS |  | 87 | -0.34 | -1.35 | 0.101 | 0.254 | 0.928 | 3595 | tags=15%, list=10%, signal=17% |
| 55 | KEGG\_JAK\_STAT\_SIGNALING\_PATHWAY |  | 151 | -0.40 | -1.35 | 0.064 | 0.252 | 0.930 | 7694 | tags=38%, list=22%, signal=49% |
| 56 | KEGG\_COLORECTAL\_CANCER |  | 62 | -0.34 | -1.35 | 0.138 | 0.249 | 0.932 | 7064 | tags=19%, list=20%, signal=24% |
| 57 | KEGG\_CYTOSOLIC\_DNA\_SENSING\_PATHWAY |  | 47 | -0.40 | -1.33 | 0.136 | 0.265 | 0.943 | 9340 | tags=38%, list=27%, signal=52% |
| 58 | KEGG\_RIBOSOME |  | 85 | -0.36 | -1.33 | 0.148 | 0.262 | 0.943 | 15827 | tags=79%, list=46%, signal=144% |
| 59 | KEGG\_NITROGEN\_METABOLISM |  | 23 | -0.52 | -1.32 | 0.152 | 0.273 | 0.946 | 8041 | tags=39%, list=23%, signal=51% |
| 60 | KEGG\_ENDOCYTOSIS |  | 179 | -0.25 | -1.32 | 0.151 | 0.272 | 0.949 | 5975 | tags=12%, list=17%, signal=14% |
| 61 | KEGG\_THYROID\_CANCER |  | 29 | -0.37 | -1.31 | 0.164 | 0.281 | 0.952 | 8828 | tags=31%, list=25%, signal=42% |
| 62 | KEGG\_SYSTEMIC\_LUPUS\_ERYTHEMATOSUS |  | 56 | -0.53 | -1.31 | 0.178 | 0.284 | 0.954 | 6840 | tags=50%, list=20%, signal=62% |
| 63 | KEGG\_RENAL\_CELL\_CARCINOMA |  | 66 | -0.42 | -1.30 | 0.157 | 0.284 | 0.956 | 10515 | tags=41%, list=30%, signal=59% |
| 64 | KEGG\_PURINE\_METABOLISM |  | 151 | -0.31 | -1.29 | 0.159 | 0.295 | 0.964 | 7922 | tags=23%, list=23%, signal=30% |
| 65 | KEGG\_VEGF\_SIGNALING\_PATHWAY |  | 75 | -0.34 | -1.29 | 0.108 | 0.294 | 0.965 | 4765 | tags=17%, list=14%, signal=20% |
| 66 | KEGG\_DRUG\_METABOLISM\_CYTOCHROME\_P450 |  | 62 | -0.38 | -1.29 | 0.091 | 0.292 | 0.966 | 4467 | tags=24%, list=13%, signal=28% |
| 67 | KEGG\_ERBB\_SIGNALING\_PATHWAY |  | 86 | -0.30 | -1.28 | 0.156 | 0.297 | 0.968 | 4956 | tags=15%, list=14%, signal=18% |
| 68 | KEGG\_FC\_GAMMA\_R\_MEDIATED\_PHAGOCYTOSIS |  | 92 | -0.32 | -1.27 | 0.155 | 0.302 | 0.969 | 10286 | tags=33%, list=30%, signal=46% |
| 69 | KEGG\_HUNTINGTONS\_DISEASE |  | 158 | -0.23 | -1.27 | 0.165 | 0.306 | 0.971 | 7334 | tags=11%, list=21%, signal=14% |
| 70 | KEGG\_MELANOMA |  | 71 | -0.36 | -1.27 | 0.125 | 0.304 | 0.972 | 959 | tags=8%, list=3%, signal=9% |
| 71 | KEGG\_LEUKOCYTE\_TRANSENDOTHELIAL\_MIGRATION |  | 114 | -0.33 | -1.26 | 0.116 | 0.308 | 0.974 | 7557 | tags=27%, list=22%, signal=35% |
| 72 | KEGG\_GLUTATHIONE\_METABOLISM |  | 49 | -0.38 | -1.26 | 0.232 | 0.311 | 0.974 | 12145 | tags=47%, list=35%, signal=72% |
| 73 | KEGG\_LINOLEIC\_ACID\_METABOLISM |  | 28 | -0.47 | -1.25 | 0.159 | 0.317 | 0.975 | 4297 | tags=29%, list=12%, signal=33% |
| 74 | KEGG\_PATHOGENIC\_ESCHERICHIA\_COLI\_INFECTION |  | 54 | -0.28 | -1.24 | 0.202 | 0.324 | 0.978 | 3512 | tags=11%, list=10%, signal=12% |
| 75 | KEGG\_METABOLISM\_OF\_XENOBIOTICS\_BY\_CYTOCHROME\_P450 |  | 60 | -0.39 | -1.24 | 0.176 | 0.321 | 0.978 | 7924 | tags=42%, list=23%, signal=54% |
| 76 | KEGG\_NON\_SMALL\_CELL\_LUNG\_CANCER |  | 54 | -0.26 | -1.23 | 0.196 | 0.337 | 0.978 | 398 | tags=4%, list=1%, signal=4% |
| 77 | KEGG\_ARGININE\_AND\_PROLINE\_METABOLISM |  | 52 | -0.34 | -1.22 | 0.216 | 0.346 | 0.983 | 6999 | tags=23%, list=20%, signal=29% |
| 78 | KEGG\_CARDIAC\_MUSCLE\_CONTRACTION |  | 72 | -0.32 | -1.21 | 0.116 | 0.353 | 0.986 | 5868 | tags=21%, list=17%, signal=25% |
| 79 | KEGG\_COMPLEMENT\_AND\_COAGULATION\_CASCADES |  | 69 | -0.37 | -1.20 | 0.170 | 0.363 | 0.988 | 5400 | tags=32%, list=16%, signal=38% |
| 80 | KEGG\_ARACHIDONIC\_ACID\_METABOLISM |  | 57 | -0.36 | -1.20 | 0.166 | 0.365 | 0.988 | 2849 | tags=16%, list=8%, signal=17% |
| 81 | KEGG\_PRIMARY\_BILE\_ACID\_BIOSYNTHESIS |  | 16 | -0.52 | -1.19 | 0.231 | 0.365 | 0.988 | 8339 | tags=50%, list=24%, signal=66% |
| 82 | KEGG\_PHENYLALANINE\_METABOLISM |  | 17 | -0.43 | -1.19 | 0.202 | 0.362 | 0.988 | 2046 | tags=18%, list=6%, signal=19% |
| 83 | KEGG\_NOTCH\_SIGNALING\_PATHWAY |  | 47 | -0.32 | -1.19 | 0.264 | 0.358 | 0.988 | 11483 | tags=43%, list=33%, signal=63% |
| 84 | KEGG\_TYPE\_II\_DIABETES\_MELLITUS |  | 47 | -0.40 | -1.17 | 0.266 | 0.390 | 0.992 | 2632 | tags=15%, list=8%, signal=16% |
| 85 | KEGG\_ENDOMETRIAL\_CANCER |  | 52 | -0.29 | -1.16 | 0.289 | 0.394 | 0.993 | 7064 | tags=19%, list=20%, signal=24% |
| 86 | KEGG\_NATURAL\_KILLER\_CELL\_MEDIATED\_CYTOTOXICITY |  | 125 | -0.31 | -1.16 | 0.203 | 0.396 | 0.993 | 6638 | tags=26%, list=19%, signal=32% |
| 87 | KEGG\_HISTIDINE\_METABOLISM |  | 28 | -0.43 | -1.16 | 0.272 | 0.393 | 0.993 | 6183 | tags=29%, list=18%, signal=35% |
| 88 | KEGG\_PYRUVATE\_METABOLISM |  | 40 | -0.30 | -1.15 | 0.304 | 0.399 | 0.993 | 11620 | tags=35%, list=33%, signal=53% |
| 89 | KEGG\_LYSINE\_DEGRADATION |  | 39 | -0.36 | -1.15 | 0.350 | 0.396 | 0.993 | 11134 | tags=38%, list=32%, signal=57% |
| 90 | KEGG\_VASCULAR\_SMOOTH\_MUSCLE\_CONTRACTION |  | 111 | -0.32 | -1.15 | 0.234 | 0.396 | 0.994 | 7850 | tags=27%, list=23%, signal=35% |
| 91 | KEGG\_ALDOSTERONE\_REGULATED\_SODIUM\_REABSORPTION |  | 42 | -0.34 | -1.14 | 0.268 | 0.404 | 0.994 | 2527 | tags=14%, list=7%, signal=15% |
| 92 | KEGG\_GLYCOLYSIS\_GLUCONEOGENESIS |  | 61 | -0.32 | -1.14 | 0.312 | 0.406 | 0.995 | 11620 | tags=48%, list=33%, signal=71% |
| 93 | KEGG\_HEMATOPOIETIC\_CELL\_LINEAGE |  | 87 | -0.37 | -1.13 | 0.218 | 0.407 | 0.995 | 5905 | tags=32%, list=17%, signal=39% |
| 94 | KEGG\_ANTIGEN\_PROCESSING\_AND\_PRESENTATION |  | 79 | -0.35 | -1.13 | 0.309 | 0.413 | 0.995 | 5132 | tags=18%, list=15%, signal=21% |
| 95 | KEGG\_CALCIUM\_SIGNALING\_PATHWAY |  | 177 | -0.30 | -1.13 | 0.212 | 0.410 | 0.995 | 6698 | tags=27%, list=19%, signal=33% |
| 96 | KEGG\_ONE\_CARBON\_POOL\_BY\_FOLATE |  | 17 | -0.40 | -1.12 | 0.337 | 0.423 | 0.996 | 9649 | tags=35%, list=28%, signal=49% |
| 97 | KEGG\_BUTANOATE\_METABOLISM |  | 34 | -0.38 | -1.10 | 0.380 | 0.449 | 0.998 | 3954 | tags=15%, list=11%, signal=17% |
| 98 | KEGG\_DNA\_REPLICATION |  | 36 | -0.33 | -1.09 | 0.402 | 0.457 | 0.998 | 13341 | tags=67%, list=38%, signal=108% |
| 99 | KEGG\_B\_CELL\_RECEPTOR\_SIGNALING\_PATHWAY |  | 75 | -0.26 | -1.09 | 0.315 | 0.453 | 0.998 | 6489 | tags=20%, list=19%, signal=25% |
| 100 | KEGG\_AUTOIMMUNE\_THYROID\_DISEASE |  | 48 | -0.40 | -1.09 | 0.354 | 0.453 | 0.998 | 6046 | tags=35%, list=17%, signal=43% |
| 101 | KEGG\_OTHER\_GLYCAN\_DEGRADATION |  | 15 | -0.37 | -1.08 | 0.359 | 0.456 | 0.998 | 12281 | tags=40%, list=35%, signal=62% |
| 102 | KEGG\_BETA\_ALANINE\_METABOLISM |  | 22 | -0.38 | -1.08 | 0.357 | 0.452 | 0.998 | 4444 | tags=18%, list=13%, signal=21% |
| 103 | KEGG\_VASOPRESSIN\_REGULATED\_WATER\_REABSORPTION |  | 44 | -0.30 | -1.07 | 0.392 | 0.468 | 0.998 | 4895 | tags=11%, list=14%, signal=13% |
| 104 | KEGG\_TRYPTOPHAN\_METABOLISM |  | 39 | -0.33 | -1.07 | 0.333 | 0.467 | 0.998 | 5864 | tags=23%, list=17%, signal=28% |
| 105 | KEGG\_GLYCOSPHINGOLIPID\_BIOSYNTHESIS\_LACTO\_AND\_NEOLACTO\_SERIES |  | 26 | -0.36 | -1.06 | 0.382 | 0.472 | 0.999 | 521 | tags=12%, list=2%, signal=12% |
| 106 | KEGG\_ECM\_RECEPTOR\_INTERACTION |  | 84 | -0.34 | -1.06 | 0.384 | 0.469 | 0.999 | 8817 | tags=42%, list=25%, signal=56% |
| 107 | KEGG\_PYRIMIDINE\_METABOLISM |  | 95 | -0.24 | -1.06 | 0.402 | 0.465 | 0.999 | 12144 | tags=37%, list=35%, signal=56% |
| 108 | KEGG\_DRUG\_METABOLISM\_OTHER\_ENZYMES |  | 42 | -0.34 | -1.06 | 0.350 | 0.462 | 0.999 | 5240 | tags=24%, list=15%, signal=28% |
| 109 | KEGG\_SELENOAMINO\_ACID\_METABOLISM |  | 24 | -0.35 | -1.06 | 0.404 | 0.460 | 0.999 | 9994 | tags=29%, list=29%, signal=41% |
| 110 | KEGG\_GLYCINE\_SERINE\_AND\_THREONINE\_METABOLISM |  | 31 | -0.31 | -1.05 | 0.387 | 0.468 | 0.999 | 8290 | tags=39%, list=24%, signal=51% |
| 111 | KEGG\_FRUCTOSE\_AND\_MANNOSE\_METABOLISM |  | 32 | -0.37 | -1.03 | 0.440 | 0.501 | 1.000 | 12400 | tags=59%, list=36%, signal=92% |
| 112 | KEGG\_GLYCEROPHOSPHOLIPID\_METABOLISM |  | 70 | -0.26 | -1.02 | 0.428 | 0.510 | 1.000 | 2898 | tags=10%, list=8%, signal=11% |
| 113 | KEGG\_GLIOMA |  | 65 | -0.24 | -1.00 | 0.453 | 0.545 | 1.000 | 2820 | tags=8%, list=8%, signal=8% |
| 114 | KEGG\_PARKINSONS\_DISEASE |  | 98 | -0.24 | -1.00 | 0.483 | 0.544 | 1.000 | 129 | tags=2%, list=0%, signal=2% |
| 115 | KEGG\_VALINE\_LEUCINE\_AND\_ISOLEUCINE\_DEGRADATION |  | 43 | -0.28 | -1.00 | 0.451 | 0.541 | 1.000 | 3954 | tags=9%, list=11%, signal=10% |
| 116 | KEGG\_LONG\_TERM\_DEPRESSION |  | 67 | -0.30 | -0.98 | 0.465 | 0.566 | 1.000 | 8960 | tags=30%, list=26%, signal=40% |
| 117 | KEGG\_NICOTINATE\_AND\_NICOTINAMIDE\_METABOLISM |  | 24 | -0.37 | -0.98 | 0.486 | 0.564 | 1.000 | 3580 | tags=21%, list=10%, signal=23% |
| 118 | KEGG\_MTOR\_SIGNALING\_PATHWAY |  | 51 | -0.28 | -0.96 | 0.506 | 0.599 | 1.000 | 7732 | tags=22%, list=22%, signal=28% |
| 119 | KEGG\_VIBRIO\_CHOLERAE\_INFECTION |  | 51 | -0.23 | -0.95 | 0.541 | 0.602 | 1.000 | 3345 | tags=8%, list=10%, signal=9% |
| 120 | KEGG\_PROGESTERONE\_MEDIATED\_OOCYTE\_MATURATION |  | 85 | -0.23 | -0.95 | 0.506 | 0.601 | 1.000 | 8453 | tags=25%, list=24%, signal=33% |
| 121 | KEGG\_N\_GLYCAN\_BIOSYNTHESIS |  | 46 | -0.29 | -0.95 | 0.528 | 0.600 | 1.000 | 10703 | tags=30%, list=31%, signal=44% |
| 122 | KEGG\_GNRH\_SIGNALING\_PATHWAY |  | 100 | -0.21 | -0.94 | 0.546 | 0.608 | 1.000 | 4711 | tags=13%, list=14%, signal=15% |
| 123 | KEGG\_PPAR\_SIGNALING\_PATHWAY |  | 69 | -0.29 | -0.94 | 0.548 | 0.604 | 1.000 | 3981 | tags=16%, list=11%, signal=18% |
| 124 | KEGG\_BIOSYNTHESIS\_OF\_UNSATURATED\_FATTY\_ACIDS |  | 19 | -0.27 | -0.94 | 0.556 | 0.602 | 1.000 | 3146 | tags=11%, list=9%, signal=12% |
| 125 | KEGG\_SPLICEOSOME |  | 125 | -0.22 | -0.93 | 0.506 | 0.615 | 1.000 | 15550 | tags=57%, list=45%, signal=102% |
| 126 | KEGG\_RETINOL\_METABOLISM |  | 55 | -0.28 | -0.92 | 0.612 | 0.630 | 1.000 | 4168 | tags=18%, list=12%, signal=21% |
| 127 | KEGG\_GAP\_JUNCTION |  | 86 | -0.20 | -0.91 | 0.687 | 0.633 | 1.000 | 4711 | tags=13%, list=14%, signal=15% |
| 128 | KEGG\_GALACTOSE\_METABOLISM |  | 25 | -0.30 | -0.91 | 0.566 | 0.640 | 1.000 | 4510 | tags=16%, list=13%, signal=18% |
| 129 | KEGG\_SNARE\_INTERACTIONS\_IN\_VESICULAR\_TRANSPORT |  | 38 | -0.26 | -0.90 | 0.603 | 0.653 | 1.000 | 3082 | tags=8%, list=9%, signal=9% |
| 130 | KEGG\_PHOSPHATIDYLINOSITOL\_SIGNALING\_SYSTEM |  | 76 | -0.21 | -0.90 | 0.611 | 0.650 | 1.000 | 10039 | tags=26%, list=29%, signal=37% |
| 131 | KEGG\_BASE\_EXCISION\_REPAIR |  | 33 | -0.28 | -0.89 | 0.540 | 0.655 | 1.000 | 13110 | tags=48%, list=38%, signal=78% |
| 132 | KEGG\_AMINO\_SUGAR\_AND\_NUCLEOTIDE\_SUGAR\_METABOLISM |  | 42 | -0.25 | -0.89 | 0.577 | 0.658 | 1.000 | 10269 | tags=31%, list=30%, signal=44% |
| 133 | KEGG\_O\_GLYCAN\_BIOSYNTHESIS |  | 29 | -0.31 | -0.88 | 0.613 | 0.657 | 1.000 | 7418 | tags=28%, list=21%, signal=35% |
| 134 | KEGG\_NUCLEOTIDE\_EXCISION\_REPAIR |  | 43 | -0.20 | -0.87 | 0.675 | 0.673 | 1.000 | 12770 | tags=49%, list=37%, signal=77% |
| 135 | KEGG\_LYSOSOME |  | 117 | -0.17 | -0.86 | 0.584 | 0.683 | 1.000 | 11970 | tags=26%, list=34%, signal=39% |
| 136 | KEGG\_NEUROACTIVE\_LIGAND\_RECEPTOR\_INTERACTION |  | 270 | -0.24 | -0.86 | 0.930 | 0.687 | 1.000 | 5009 | tags=21%, list=14%, signal=24% |
| 137 | KEGG\_PROXIMAL\_TUBULE\_BICARBONATE\_RECLAMATION |  | 23 | -0.29 | -0.85 | 0.716 | 0.703 | 1.000 | 7552 | tags=30%, list=22%, signal=39% |
| 138 | KEGG\_REGULATION\_OF\_AUTOPHAGY |  | 30 | -0.28 | -0.85 | 0.660 | 0.700 | 1.000 | 6046 | tags=23%, list=17%, signal=28% |
| 139 | KEGG\_UBIQUITIN\_MEDIATED\_PROTEOLYSIS |  | 130 | -0.14 | -0.83 | 0.667 | 0.717 | 1.000 | 11536 | tags=28%, list=33%, signal=41% |
| 140 | KEGG\_AMYOTROPHIC\_LATERAL\_SCLEROSIS\_ALS |  | 53 | -0.23 | -0.83 | 0.717 | 0.712 | 1.000 | 6432 | tags=19%, list=19%, signal=23% |
| 141 | KEGG\_LONG\_TERM\_POTENTIATION |  | 70 | -0.19 | -0.83 | 0.744 | 0.709 | 1.000 | 6379 | tags=17%, list=18%, signal=21% |
| 142 | KEGG\_ALPHA\_LINOLENIC\_ACID\_METABOLISM |  | 18 | -0.35 | -0.83 | 0.662 | 0.711 | 1.000 | 4297 | tags=17%, list=12%, signal=19% |
| 143 | KEGG\_PROTEASOME |  | 43 | -0.23 | -0.81 | 0.717 | 0.728 | 1.000 | 26685 | tags=100%, list=77%, signal=431% |
| 144 | KEGG\_RNA\_POLYMERASE |  | 27 | -0.25 | -0.78 | 0.805 | 0.778 | 1.000 | 26160 | tags=100%, list=75%, signal=405% |
| 145 | KEGG\_RENIN\_ANGIOTENSIN\_SYSTEM |  | 17 | -0.33 | -0.75 | 0.797 | 0.825 | 1.000 | 7952 | tags=29%, list=23%, signal=38% |
| 146 | KEGG\_GLYOXYLATE\_AND\_DICARBOXYLATE\_METABOLISM |  | 16 | -0.25 | -0.71 | 0.809 | 0.873 | 1.000 | 12127 | tags=44%, list=35%, signal=67% |
| 147 | KEGG\_PROPANOATE\_METABOLISM |  | 31 | -0.21 | -0.67 | 0.813 | 0.910 | 1.000 | 3165 | tags=6%, list=9%, signal=7% |
| 148 | KEGG\_ABC\_TRANSPORTERS |  | 44 | -0.20 | -0.60 | 0.981 | 0.961 | 1.000 | 6297 | tags=18%, list=18%, signal=22% |
| 149 | KEGG\_PANTOTHENATE\_AND\_COA\_BIOSYNTHESIS |  | 16 | -0.22 | -0.57 | 0.959 | 0.970 | 1.000 | 7199 | tags=19%, list=21%, signal=24% |
| 150 | KEGG\_OXIDATIVE\_PHOSPHORYLATION |  | 98 | -0.13 | -0.57 | 0.901 | 0.965 | 1.000 | 6969 | tags=7%, list=20%, signal=9% |
Table: Gene sets enriched in phenotype **l (8 samples) **[plain text format]****
